# Supplementation of arachidonic acid-enriched oil increases arachidonic acid contents in plasma phospholipids, but does not increase their metabolites and clinical parameters in Japanese healthy elderly individuals: a randomized controlled study

**DOI:** 10.1186/1476-511X-10-241

**Published:** 2011-12-22

**Authors:** Saki Kakutani, Yoshiyuki Ishikura, Norifumi Tateishi, Chika Horikawa, Hisanori Tokuda, Masanori Kontani, Hiroshi Kawashima, Yutaka Sakakibara, Yoshinobu Kiso, Hiroshi Shibata, Ikuo Morita

**Affiliations:** 1 Institute for Health Care Science, Suntory Wellness Ltd., Osaka, Japan; 2 Safety Science Institute, Suntory Business Expert Ltd., Osaka, Japan; 3 Department of Cellular Physiological Chemistry, Graduate School, Tokyo Medical and Dental University, Tokyo, Japan

**Keywords:** arachidonic acid, thromboxane A_2_, prostacyclin, prostaglandin E_2_, cardiovascular diseases, inflammation

## Abstract

**Background:**

The importance of arachidonic acid (ARA) among the elderly has recently gained increased attention. The effects of ARA supplementation in the elderly are not fully understood, although ARA is considered to be associated with various diseases. We investigate whether ARA supplementation to Japanese elderly subjects affects clinical parameters involved in cardiovascular, inflammatory, and allergic diseases. We also examine the levels of ARA metabolites such as prostanoids during intervention.

**Methods:**

We conducted a randomized, double-blind and placebo-controlled parallel group intervention trial. ARA-enriched oil (240 or 720 mg ARA per day) or placebo was administered to Japanese healthy men and women aged 55-70 years for 4 weeks followed by a 4-week washout period. The fatty acid contents of plasma phospholipids, clinical parameters, and ARA metabolites were determined at baseline, 2, 4, and 8 weeks.

**Results:**

The ARA content in plasma phospholipids in the ARA-administrated groups increased dose-dependently and was almost the same at 2 weeks and at 4 weeks. The elevated ARA content decreased to nearly baseline during a 4-week washout period. During the supplementation and washout periods, no changes were observed in eicosapentaenoic acid and docosahexaenoic acid contents. There were no changes in clinical blood parameters related to cardiovascular, inflammatory and allergic diseases. ARA supplementation did not alter the level of ARA metabolites such as urinary 11-dehydro thromboxane B_2_, 2,3-dinor-6-keto prostaglandin (PG) F_1α _and 9,15-dioxo-11α-hydroxy-13,14-dihydro-2,3,4,5-tetranor-prostan-1,20-dioic acid (tetranor-PGEM), and plasma PGE_2 _and lipoxin A_4_. ARA in plasma phospholipids was not correlated with ARA metabolite levels in the blood or urine.

**Conclusion:**

These results indicate that ARA supplementation, even at a relatively high dose, does not increase ARA metabolites, and suggest that it does not induce cardiovascular, inflammatory or allergic diseases in Japanese elderly individuals.

## Background

Arachidonic acid (ARA) is an n-6 essential fatty acid that is a major constituent of biomembranes. It is converted into lipid mediators that exert various physiological actions. ARA is synthesized in the body from dietary linoleic acid (LA) and additionally most adults consume 50-250 mg/day of ARA from foodstuffs [1-3].

The consumption of ARA in breast milk is very important for infant development since the activity from LA conversion to ARA is low in infant [4]. The conversion of LA to ARA declines with age [5], and the importance of ARA supplementation among the elderly has recently gained increased attention. It has been reported that supplementation with ARA among the elderly improves cognitive response [6] and coronary flow velocity reserve [7] and some animal studies support these findings [8-11].

However, many studies show that lipid mediators derived from ARA are associated with various diseases. For example, thromboxane A_2 _(TXA_2_) is associated with cardiovascular diseases via its activation of thrombogenicity and vasoconstriction, whereas prostaglandin E_2 _(PGE_2_) leads to inflammation and might enhance tumor growth [12-14]. Levels of the urinary TXA_2 _metabolite, 11-dehydro TXB_2_, have been shown to be higher in patients with heart failure (3.4-fold), ischemic heart disease (1.4-fold) [15] and essential hypertension [16]. Plasma PGE_2 _levels are also high in patients with ulcerative colitis [17] and advanced periodontitis [18], and levels of its urinary metabolite, tetranor-PGEM, are epidemiologically higher in colorectal cancer [19]. The relationship between lipids mediators and diseases is speculated based on the fact that cyclooxygenase (COX) inhibitors are effective against these conditions [14,20].

Many clinical trials of ARA supplementation have been done on infants [4], but there are a few reports describe the administration of ARA or ARA-containing oil to adults [21-23]. Among healthy males who consumed 1.5 g/day of ARA (as free ARA) for 50 days [21,22] or 838 mg/day of ARA for 4 weeks [23] in randomized controlled studies, platelet aggregation did not change and adverse effects did not occur. Both urinary 11-dehydro TXB_2 _and 2,3-dinor-6-keto PGF_1α _slightly increased in the former study [22]. However, it remains unclear whether ARA intake evokes the clinical parameters in the speculated diseases in the elderly. It is also unclear whether ARA intake increases lipid mediators derived from ARA in the elderly.

The present study investigates the effects of 240 or 720 mg/day of ARA, which is much more than that derived from food, on Japanese healthy elderly individuals. We determined clinical parameters of cardiovascular, inflammatory, and allergic diseases in blood as well as ARA content in plasma phospholipids and urinary and plasma lipid mediators. Correlations between ARA content in plasma phospholipids and lipid mediator concentrations were also determined.

## Materials and methods

### Study design

This randomized, double-blind and placebo-controlled parallel group intervention trial evaluated the effects of daily ARA supplementation on cardiovascular disease and/or inflammation. The Ethics Committee on Human Experimentation of Suntory Holdings Ltd. approved the study, which conformed to the principles set forth in the Declaration of Helsinki. Written informed consent was obtained from the participants of this study. Physiological parameters and blood and urine were sampled at the time of recruitment starting in August 2010. One hundred and eighteen participants were screened and randomly assigned to placebo, low-ARA or high-ARA groups. Participants received 10 gelatin-capsules containing either ARA or a placebo every morning for 4 weeks followed by a 4-week washout period. Blood and urine were sampled, a study diary was distributed and collected and dietary intake was assessed at baseline (week 0, within 4 weeks of recruitment) and again at 2, 4 and 8 weeks later. The fatty acid composition of the oils used in this study is shown in Table 1. The placebo group consumed 1700 mg/day of commercially available olive oil. The high-ARA group consumed 1700 mg/day of an ARA-enriched edible oil derived from *Mortierella alpina *(SUNTGA40S; 720 mg/day of ARA) [24]. The low-ARA group received 570 mg/day of ARA-enriched oil and 1130 mg/day of olive oil (240 mg/day of ARA). Blood and urine samples were obtained after an overnight fast for > 10 hours on the morning of each assessment. Cardiovascular risk parameters included prothrombin time (PT), activated partial thromboplastin time (APTT), antithrombin III (ATIII), high-sensitivity C-reactive protein (hs-CRP) and adiponectin. Allergic parameters included nonspecific immunoglobulin E (IgE) levels and eosinophils (EO). Inflammatory parameters comprised C-reactive protein (CRP), interleukin-6 (IL-6) and tumor necrosis factor-α (TNF-α) levels. Urinalysis was conducted for quantitative analysis of creatinine (Cre) and qualitative analyses of protein, glucose and urobilinogen.

**Table 1:** Fatty acid composition of test capsules


**Fatty acids^1^**	**Group**		
**(%)**	**Placebo**	**Low-ARA**	**High-ARA**

14:0	0.0	0.2	0.5
15:0	0.0	0.0	0.2
16:0	12.2	11.9	11.3
16:1	1.2	0.8	0.0
17:0	0.0	0.1	0.3
18:0	2.8	4.5	8.1
18:1	72.3	50.3	6.3
18:2n-6	9.6	9.4	8.9
18:3n-6	0.0	0.9	2.6
18:3n-3	0.7	0.6	0.4
20:0	0.4	0.6	0.9
20:1	0.3	0.3	0.4
20:2n-6	0.0	0.2	0.7
20:3n-6	0.0	1.4	4.0
20:4n-6	0.0	14.2	42.9
22:0	0.1	1.2	3.3
22:4n-6	0.0	0.2	0.5
24:0	0.0	2.6	7.5
Others	0.4	0.6	1.2

^1^14:0, myristic acid; 15:0, pentadecanoic acid; 16:0; palmitic acid; 16:1, palmitoleic acid; 17:0, heptadecanoic acid; 18:0, stearic acid; 18:1, oleic acid; 18:2n-6, linoleic acid; 18:3n-6, γ-linolenic acid; 18:3n-3, α-linolenic acid; 20:0, eicosanoic acid; 20:1, eicosenic acid; 20:2n-6, eicosadienoic acid; 20:3n-6, dihomo-γ-linolenic acid; 20:4n-6, arachidonic acid; 22:0, behenic acid; 22:4n-6, docosatetraenoic acid; and 24:0, lignoceric acid.

### Participants

Healthy men and women aged 55-70 years living in Tokyo and its environs were recruited. Exclusion criteria were as follows: allergy to gelatin or olive oil; continuous consumption of drugs or supplements that affect lipid metabolism; continuous intake of non-steroidal anti-inflammatory or anti-allergic drugs; a history of serious disorders such as cardiac infarction, cerebral infarction, stroke, cancer, asthma; and clinically significant systemic diseases. Participants were randomly assigned to the three groups matched by gender, age, hs-CRP, PT and estimated ARA content (%) in plasma phospholipids. To quickly estimate the ARA content in plasma phospholipids at the recruitment, the estimated ARA content was calculated from triglycerides (TG, mg/dL), phospholipids (PL, mg/dL) and the ARA content in total plasma fatty acids as: (ARA content in total plasma fatty acids)×(TG + PL)/PL. This rough estimation is based on the fact that the ARA content is much smaller in plasma TG than in plasma PL (the ARA in plasma TG is negligibly-small for the rough estimation), and used only for quick assignment to the three groups.

### Chemicals and apparatus

We purchased 11-dehydro TXB_2_, 9,15-dioxo-11α-hydroxy-13,14-dihydro-2,3,4,5-tetranor-prostan-1,20-dioic acid (tetranor-PGEM), lipoxin A_4 _(LXA_4_), 11-dehydro TXB_2_-d4, 9,15-dioxo-11α-hydroxy-2,3,4,5-tetranor-prostan-1,20-dioic-13,13,14,14,15,15-d6 acid (tetranor-PGEM-d6), and LXA_4_-d5 from Cayman Chemical (Ann Arbor, MI, USA). Fatty acid methyl esters were chromatographically separated and detected using an Agilent 6890 GLC system (Agilent Technologies, Santa Clara, CA, USA) equipped with a Supelco SP-2330 column (30 m × 0.32 mm × 0.2 μm, Sigma-Aldrich, St. Louis, MO, USA). Lipid mediators were chromatographically separated and detected using an Agilent 1200 HPLC system (Agilent Technologies) equipped with a Cadenza CD-C18 column (3 μm, 2 mm i.d. × 150 mm, Imtakt, Kyoto, Japan) and a 4000 Q TRAP with electrospray interface (AB SCIEX, Foster City, CA, USA).

### Fatty acid analysis

Lipids in plasma were extracted and purified by the method of Folch et al. [25]. Fatty acid residues in lipid fractions were analyzed by the method of Sakuradani et al. [26]. In brief, each fraction was incubated with an internal standard (pentadecanoic acid) in methanolic HCl at 50°C for 3 h to transmethylate fatty acid residues to fatty acid methyl esters, which were extracted with *n*-hexane and analyzed by capillary gas-liquid chromatography. Plasma was directly transmethylated without extraction or fractionation when we calculated the estimated ARA content in plasma phospholipids.

### Analysis of urinary metabolites of lipid mediators

Urine samples were stored at -80°C for 5-41 days before measurement of metabolites. 

Urinary 11-dehydro TXB_2 _was measured by LC-MS/MS. Urine samples (0.5 mL), to which 11-dehydro TXB_2_-d4 was added as an internal standard, were diluted with 1 mmol/L HCl to a final volume of approximately 3 mL and left for 1 h at room temperature. The mixtures were applied to preconditioned SPE cartridges (Empore disk cartridge C18-SD, 3M, St. Paul, MN, USA), and the cartridges were washed with 1 mmol/L HCl, water and hexane. 11-Dehydro TXB_2 _and 11-dehydro TXB_2_-d4 were then eluted with 1 mL of hexane/ethyl acetate (1/1, v/v). The eluates were dried by centrifugal evaporation, re-dissolved in 0.1 mL of acetonitrile/water/formic acid (250/750/1, v/v/v) and then transferred to brown glass vials at 10°C. Portions of these elute (20 μL) were injected into LC-MS/MS. Solvent A was 5 mM ammonium acetate (pH 5.5) and solvent B was acetonitrile. The separation was performed in an isocratic mode with 35% solvent B at a flow rate of 0.2 mL/min and a column temperature of 40°C. The mass spectrometer was operated in the negative ion mode. 11-Dehydro TXB_2 _and 11-dehydro TXB_2_-d4 were detected in selected reaction monitoring (SRM) mode by monitoring mass transitions of *m/z *367 → 305 for 11-dehydro TXB_2_, and *m/z *371 → 309 for 11-dehydro TXB_2_-d4 at a collision energy of -23 V.

Urinary tetranor-PGEM was measured by LC-MS/MS according to the modified method of Murphey et al. [27]. Urine samples (0.1 mL) to which tetranor-PGEM-d6 was added as an internal standard were diluted with 1 mmol/L HCl to a final volume of approximately 1 mL. The diluted samples were mixed with 0.5 mL of *O*-methylhydroxylamine hydrochloride in 1.5 M sodium acetate buffer pH 5 (16%, w/v) and left for 1 h at room temperature. The mixtures were applied to SPE cartridges as described above, and the cartridges were washed with 1 mmol/L HCl. Tetranor-PGEM and tetranor-PGEM-d6 were then eluted with 1 mL of ethyl acetate. The eluates were dried, re-solved and transferred as described above. Portions of these elute (20 μL) were injected into LC-MS/MS. Solvent A was water/formic acid (100/0.2, v/v) and solvent B was acetonitrile/methanol/formic acid (95/5/0.2, v/v/v). The separation was performed at a flow rate of 0.2 mL/min and a column temperature of 60°C using the following linear gradient: 0-4.8 min, 30 to 94% solvent B; 4.8-5.75 min, 94 to 30% solvent B; 5.75-14.8 min, 30% solvent B. The mass spectrometer was operated in the negative ion mode. Tetranor-PGEM and tetranor-PGEM-d6 were detected in SRM mode by monitoring mass transitions at *m/z *385 → 336 for tetranor-PGEM, and *m/z *391 → 342 for tetranor-PGEM-d6 at a collision energy of -25 V.

Urinary 2,3-dinor-6-keto PGF_1α _was measured using an enzyme-linked immunosorbent assay (EIA) kit (2,3-dinor-6-keto Prostaglandin F_1α _EIA Kit, Cayman Chemical Company). Urine samples (0.5 mL) were diluted with 1 M sodium citrate buffer pH 4 to a final volume of approximately 1 mL and then vigorously mixed with 4 mL of ethyl acetate. The mixtures were separated by centrifugation and the upper phases were collected. The liquid-liquid extraction with ethyl acetate was performed three times and three upper phases of one urine sample were pooled. The pooled extracts were dried by centrifugal evaporation and re-dissolved in assay buffer for analysis.

### Analysis of plasma lipid mediators

Blood samples for lipid mediators were collected in vacuum blood collection tubes containing EDTA-2Na and a final concentration of approximately 18 μM of sodium indomethacin. Plasma separated within 1 h was then stored at -80°C for 10-93 days before measurement of mediators.

Plasma PGE_2 _was measured using an EIA kit (Prostaglandin E_2 _EIA Kit - Monoclonal, Cayman Chemical Company). Plasma samples (1 mL) to which 5 μL of formic acid was added were diluted with 1 mmol/L HCl to a final volume of approximately 3 mL. The mixtures were applied to preconditioned SPE cartridges (BondElut C18, Agilent Technologies), and the cartridges were washed with 1 mmol/L HCl and hexane. PGE_2 _was then eluted with 1 mL of ethyl acetate/methanol (99/1, v/v). The eluates were dried by centrifugal evaporation and re-dissolved in assay buffer for analysis.

Plasma LXA_4 _was measured by LC-MS/MS. Plasma samples (1 mL) to which LXA_4_-d5 was added as an internal standard were diluted with water/acetic acid (1000/5, v/v) to a final volume of approximately 3 mL and applied to preconditioned SPE cartridges (FOCUS, 20 mg/3 mL, Agilent Technologies). The cartridges were washed with water/acetic acid (1000/5, v/v) and water. LXA_4 _and LXA_4_-d5 were then eluted with 1 mL of methanol/acetonitrile/acetic acid (600/300/1, v/v/v). The eluates were dried, re-solved and transferred as described above. Portions of these elute (20 μL) were injected into LC-MS/MS. Solvent A was water/formic acid (100/0.2, v/v) and solvent B was acetonitrile/methanol/formic acid (95/5/0.2, v/v/v). The separation was performed at a flow rate of 0.2 mL/min and a column temperature of 60°C using the following linear gradient: 0-3.75 min, 50 to 98% solvent B; 3.75-5 min, 98% solvent B; 5-5.75 min, 98 to 50% solvent B; 5.75-14.8 min, 50% solvent B. The mass spectrometer was operated in the negative ion mode. LXA_4 _and LXA_4_-d5 were detected in SRM mode by monitoring mass transitions at *m/z *351 → 115 for LXA_4_, and *m/z* 356 → 115 for LXA_4_-d5 at a collision energy of -22 V.

### Dietary assessment and study diary

Dietary habits during the preceding month were assessed using the brief self-administered diet history questionnaire (BDHQ) [28]. Dietary intake was estimated using an ad hoc computer algorithm for the BDHQ based on the Standard Tables of Food Composition in Japan [29,30]. Participants were asked to keep a record throughout the study about intake of the test capsules, the presence of symptoms, amount of exercise, amount of food and alcohol consumed and the use of medication.

### Statistical analysis

Results are expressed as means ± SD. Hs-CRP values that exceeded the upper limit of detection (1000 μg/dL) were rounded down to 1000 μg/dL and comprised of one measurement in the low-ARA group at baseline, one in the placebo group, one in the low-ARA group at 2 weeks and two in the high-ARA group at 4 weeks.

For physiological parameters, blood biochemical parameters except hs-CRP, hematological parameters, fatty acid composition of plasma phospholipids, urinary metabolites of lipid mediators and plasma lipid mediators, intra-group comparisons at 2, 4 or 8 weeks versus baseline were analyzed by repeated ANOVA and Dunnett's test using the actual values; inter-group comparisons at 2, 4, or 8 weeks were analyzed by ANOVA and the Tukey-Kramer test using the changes from baseline values. For hs-CRP, intra-group comparisons were analyzed by Friedman test and Steel test; inter-group comparisons were analyzed by Kruskal-Wallis test and Steel-Dwass test. For dietary intake of nutrients, intra-group comparisons at 4 weeks versus baseline were analyzed by a paired student t-test; inter-group comparisons at 4 weeks were analyzed by ANOVA and Tukey-Kramer test. For compliance rate, inter-group comparisons were analyzed by ANOVA and Tukey-Kramer test. For adverse events, inter-group comparisons were analyzed by Kruskal-Wallis test. All *p* values were two-tailed, and a *p* value of < 0.05 was considered statistically significant.

## Results

### Characteristics of the participants

**Figure 1:**
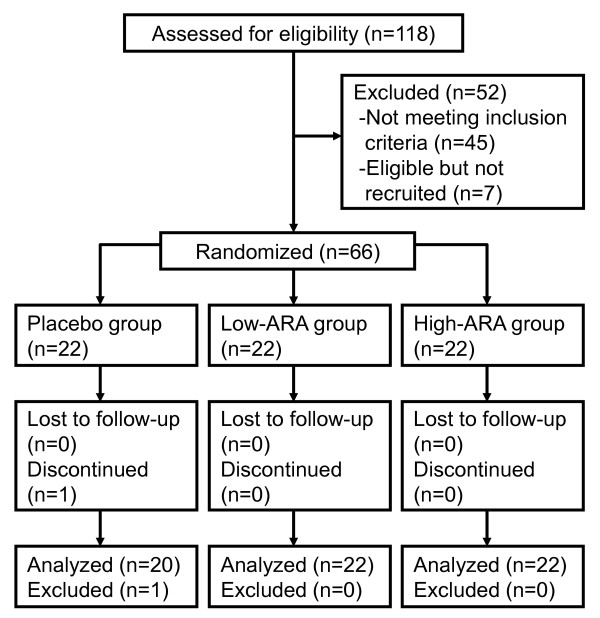
**Flow diagram of participants included in the present analysis**.

One participant in the placebo group withdrew for personal reasons and another was excluded due to meeting one of the exclusion criteria. Thus, we analyzed data generated from 64 participants in three groups (placebo, n = 20; low-ARA, n = 22; and high-ARA, n = 22) (Figure 1). The mean compliance rate was > 95% across the three groups and did not differ significantly among them. Side effects did not arise. The numbers of adverse events that developed were six among five participants in the placebo group, eight among six in the low-ARA group and twelve among six in the high-ARA group. None of these adverse events were severe and their frequency did not significantly differ among the groups. The adverse events were common cold (all groups), eczema (placebo and low-ARA groups), diarrhoea (high-ARA group), toothache (low-ARA group), and bone fracture (placebo group).

Baseline characteristics of the three groups are shown in Table 2. All groups were balanced with respect to gender, age, BMI, alcohol consumption, smoking status and exercise. Neither hs-CRP, PT nor cardiovascular risk parameters differed significantly among the groups. The mean ARA content in plasma phospholipids ranged from 8.2-8.8% among the three groups and other polyunsaturated fatty acids also did not differ among them. Macronutrient intake during the preceding month at baseline or at 4 weeks later did not differ among the groups or at any time point (Table 3). The daily ARA intake derived from food ranged from 170-200 mg/day in all three groups with no significant differences. The daily intakes of DHA and EPA were 300-500 mg/day and 500-800 mg/day, respectively.

**Table 2:** Baseline characteristics of the participants^1^


			**Group**	
**Characteristics^2^**		**Placebo**	**Low-ARA**	**High-ARA**
		**(n = 20)**	**(n = 22)**	**(n = 22)**

Gender (Female)	n	12	13	12
Age	y	63.1 ± 3.8	62.8 ± 4.3	62.9 ± 4.2
BMI	kg/m^2^	21.5 ± 2.4	22.5 ± 2.0	22.5 ± 3.1
Alcohol consumption				
Positive	n	11	13	9
Negative	n	9	9	13
Smoking status				
Positive	n	4	3	3
Negative	n	16	19	19
Exercise				
Habitual	n	11	15	14
Nonhabitual	n	9	7	8
hs-CRP	μg/dL	113 ± 98	106 ± 153	104 ± 116
PT	%	91.4 ± 8.5	91.4 ± 8.6	91.5 ± 6.5
HbA1c	%	4.9 ± 0.2	4.9 ± 0.3	4.9 ± 0.4
FA composition				
18:2n-6	%	18.33 ± 2.64	18.91 ± 2.16	17.78 ± 2.62
20:4n-6	%	8.27 ± 1.26	8.61 ± 0.92	8.77 ± 1.32
20:5n-3	%	2.85 ± 1.30	3.17 ± 1.42	3.59 ± 2.16
22:6n-3	%	7.83 ± 1.42	8.10 ± 1.27	8.41 ± 2.29

^1^Values are means ± SD. No significance among the groups (ANOVA and Tukey-Kramer or Kruskal-Wallis tests).

^2^hs-CRP, high-sensitivity CRP; PT, prothrombin time; HbA1c, hemoglobin A1c; FA, fatty acid; 18:2n-6, linoleic acid; 20:4n-6, arachidonic acid; 20:5n-3, eicosapentaenoic acid; 22:6n-3, docosahexaenoic acid.

**Table 3:** Calculated daily nutrient intake at baseline and after four weeks of supplementation ^1^


**Nutrient**		**Baseline (week 0)**			**Supplementation (week 4)**		
		**Placebo**	**Low-ARA**	**High-ARA**	**Placebo**	**Low-ARA**	**High-ARA**

Energy	kcal/d	1871 ± 483	1779 ± 439	2017 ± 636	1973 ± 550	1748 ± 410	1945 ± 497
Protein	g/d	73.4 ± 21.6	67.1 ± 17.2	78.0 ± 22.2	74.6 ± 21.4	68.9 ± 17.8	77.0 ± 21.4
Carbohydrate	g/d	256 ± 68	234 ± 82	260 ± 91	278 ± 76	227 ± 63	259 ± 74
Total fat	g/d	56.0 ± 19.1	52.8 ± 16.6	61.0 ± 21.9	56.5 ± 22.3	52.4 ± 14.8	57.5 ± 16.8
SFA	g/d	16.2 ± 6.3	14.7 ± 5.0	16.3 ± 6.7	15.5 ± 8.1	14.1 ± 4.4	15.2 ± 5.2
MUFA	g/d	19.3 ± 7.0	18.4 ± 6.4	21.8 ± 8.2	19.6 ± 7.6	18.4 ± 5.7	20.4 ± 6.1
PUFA	g/d	13.1 ± 4.6	12.7 ± 3.6	14.8 ± 4.7	13.8 ± 4.3	12.8 ± 3.4	14.1 ± 4.0
18:2n-6	g/d	9.96 ± 3.52	9.76 ± 2.85	11.12 ± 3.60	10.59 ± 3.34	9.71 ± 2.56	10.74 ± 3.05
20:4n-6	mg/d	174 ± 56	170 ± 71	199 ± 74	172 ± 67	174 ± 73	189 ± 66
18:3n-3	g/d	1.61 ± 0.61	1.52 ± 0.48	1.79 ± 0.62	1.67 ± 0.57	1.51 ± 0.41	1.71 ± 0.51
20:5n-3	mg/d	377 ± 215	315 ± 172	447 ± 163	366 ± 197	367 ± 190	399 ± 202
22:6n-3	mg/d	616 ± 323	538 ± 267	729 ± 261	600 ± 289	611 ± 301	658 ± 306

^1^Values are means ± SD (n = 20, placebo group; n = 22, low-ARA; n = 22, high-ARA). No significant differences between time points (Student's t-test) or groups (ANOVA and Tukey-Kramer test).

^2^SFA, saturated fatty acid; MUFA, monounsaturated fatty acid; PUFA, polyunsaturated fatty acid; 18:2n-6, linoleic acid; 20:4n-6, arachidonic acid; 18:3n-3, α-linolenic acid; 20:5n-3, eicosapentaenoic acid; and 22:6n-3, docosahexaenoic acid.

### Fatty acid profiles of plasma phospholipids

**Figure 2:**
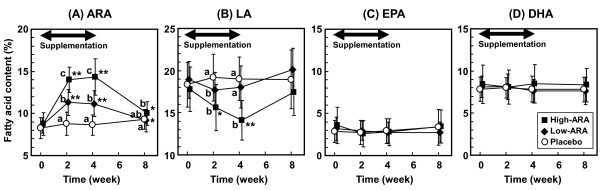
**Fatty acid content in plasma phospholipids ((A) ARA, (B) LA, (C) DHA and (D) EPA) during 4-week supplementation and 4-week washout**. Placebo, low-ARA and high-ARA groups are indicated by open circles, closed diamonds and closed squares, respectively. Values are means ± SD (n = 20 (placebo) and n = 22 (in each low- and high-ARA group)). *p < 0.05, **p < 0.01 vs. baseline in group (repeated ANOVA and Dunnett's test). ^#^*p *< 0.05 vs. placebo group and ^$^*p *< 0.05 vs. low-ARA group when amount of change from baseline significantly differed at each time point (ANOVA and Tukey-Kramer test).

The ARA content in plasma phospholipids in the high-ARA group increased from 8.77 ± 1.32% (means ± SD) at baseline to 14.02 ± 1.50% at 2 weeks, and was almost the same at 4 weeks (14.33 ± 2.14%). The elevated ARA content declined almost to the initial level during the 4-week washout period (10.00 ± 1.39%) (Figure 2A). The time course of the ARA increase tended to be similar in the low-ARA group. The ARA content in the low-ARA group was 8.61 ± 0.92% at baseline, 11.30 ± 1.55% at 2 weeks, and 11.15 ± 1.52% at 4 weeks; the value declined during the washout period. The ARA content in the placebo group remained unchanged throughout the study.

In the high-ARA group, the LA content in plasma phospholipids inversely declined from 17.78 ± 2.62% at baseline to 14.10 ± 2.36% at 4 weeks and then increased almost to baseline levels during the 4 week washout period (17.41 ± 2.73%) (Figure 2B). The EPA and DHA contents in plasma phospholipids were unchanged throughout the study period in all groups (Figure 2C and 2D).

### Clinical parameters of cardiovascular diseases and inflammation

**Figure 3:**
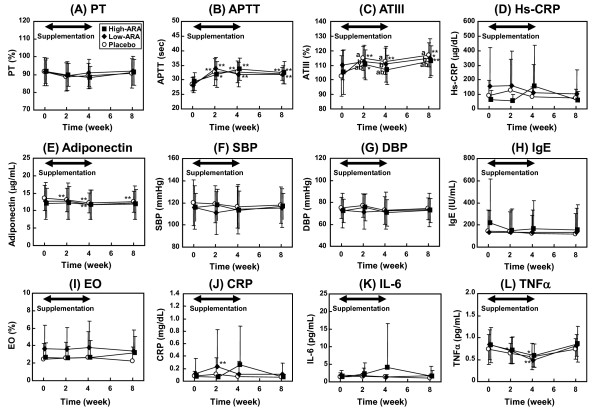
**Plasma parameters for cardiovascular (A-G), allergic (H-I) and inflammatory (J-L) diseases during 4-week supplementation and 4-week washout**. Placebo, low-ARA and high-ARA groups are indicated by open circles, closed diamonds and closed squares, respectively. Values are means ± SD (n = 20 (placebo) and n = 22 (in each low- and high-ARA group)). *p < 0.05, **p < 0.01 vs. baseline in group (ANOVA and Dunnett's test). Values without a common letter are significantly different at *p *< 0.05 (ANOVA and Tukey-Kramer test).

Changes in clinical parameters associated with cardiovascular risk, allergy and inflammation are shown in Figure 3. Although some parameters the groups or time points differed significantly, all values were within the normal ranges and did not change according to ARA supplementation. Physiological, blood biochemical and hematological parameters were also within normal ranges and were unaffected by dose of ARA (Additional file 1, Table S1). Urinary findings were normal in all groups (data not shown).

### Levels of ARA metabolites in urine and blood

**Figure 4:**
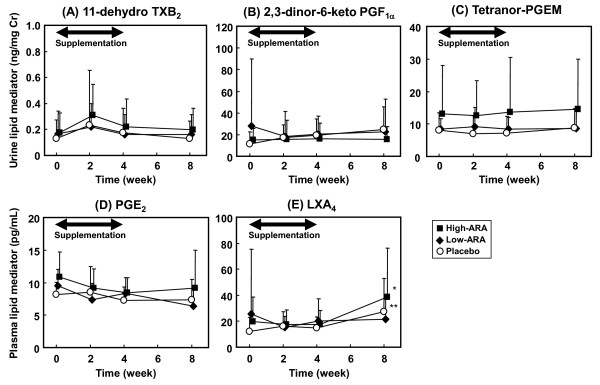
**Change in ARA metabolites during 4-week supplementation and 4-week washout**. (A) Urinary 11-dehydro TXB_2_, (B) urinary 2,3-dinor-6-keto- PGF_1α_, (C) urinary tetranor-PGEM, (D) plasma PGE_2 _and (E) plasma LXA_4_. Placebo, low-ARA and high-ARA groups are indicated by open circles, closed diamonds and closed squares, respectively. Values are means ± SD (n = 20 (placebo) and n = 22 (in each low- and high-ARA group)). *p < 0.05, **p < 0.01 vs. baseline in group (ANOVA and Dunnett's test). Values without a common letter are significantly different at *p *< 0.05 (ANOVA and Tukey-Kramer test).

Concentrations of ARA metabolites in urine and blood are shown in Figure 4. Initial concentrations of urinary metabolites in the placebo, the low-ARA and the high-ARA groups were not significantly different. After the supplementation for 4 weeks, none of the urinary metabolites in the low-ARA and high-ARA groups significantly increased from initial levels; there were no differences among the groups at 2 and 4 weeks. At 4 weeks, the concentrations of 11-dehydro TXB_2_, 2,3-dinor-6-keto PGF_1α _and tetranor-PGEM in the low-ARA group were 0.160 ± 0.156 (Figure 4A), 20.6 ± 16.7 (Figure 4B) and 8.41 ± 3.63 ng/mg Cre, respectively (Figure 4C); those in high-ARA group were 0.222 ± 0.215 (Figure 4A), 16.4 ± 14.1 (Figure 4B) and 16.4 ± 14.1 ng/mg Cre, respectively (Figure 4C).

Plasma ARA-metabolites were also unchanged from the initial levels during the supplementation and showed no differences at 2 and 4 weeks among the three groups. The initial concentrations of PGE_2 _in the placebo, the low-ARA and the high-ARA groups were 8.12 ± 1.96, 9.52 ± 2.48 and 10.9 ± 3.79 pg/mL, respectively (Figure 4D), and those of LXA_4 _were 11.9 ± 10.8, 25.4 ± 50.0 and 19.9 ± 18.7 pg/mL, respectively (Figure 4E), respectively. After the supplementation for 4 weeks, the concentrations of PGE_2 _and LXA_4 _in the low-ARA group were 8.37 ± 2.40 (Figure 4C) and 20.4 ± 17.1 pg/mL, respectively (Figure 4E); those in the high-ARA group were 8.44 ± 2.33 (Figure 4C) and 17.7 ± 10.7 pg/mL, respectively (Figure 4E).

**Figure 5:**
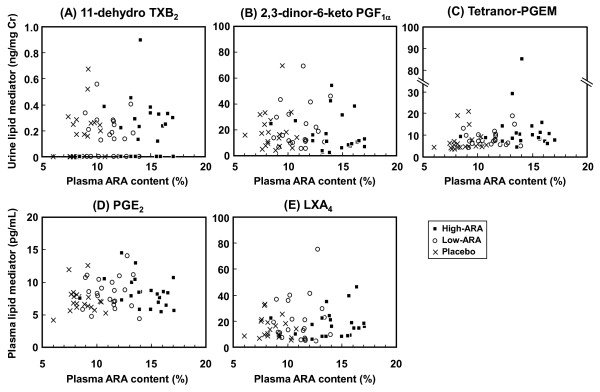
**Correlation between plasma ARA content and ARA metabolites in blood and urine**. (A) Urinary 11-dehydro TXB_2_, (B) urinary 2,3-dinor-6-keto- PGF_1α_, (C) urinary tetranor-PGEM, (D) plasma PGE_2 _and (E) plasma LXA_4_ after 4-week supplementation. Placebo, low-ARA and high-ARA groups are shown as crosses (n = 20), open circles, (n = 22) and closed squares (n = 22). No correlations were identified in any of these analyses (A)-(E).

None of the ARA metabolites measured in urine and blood measured was correlated with ARA contents in plasma phospholipids at 4 weeks of ARA supplementation (Figure 5).

## Discussion

The present study clarified that in spite of the increase in ARA levels in plasma phospholipids, plasma clinical parameters of cardiovascular, inflammatory and allergic diseases and levels of ARA metabolites in urine and blood were not altered among the healthy elderly participants whose diet was supplemented with an ARA-enriched oil (240 or 720 mg/day ARA) for 4 weeks.

In previous studies, ARA content in plasma phospholipids increased by 0.7% in young females supplemented with 80 mg/day of ARA for 3 weeks [31], 2.0% in elderly males given 240 mg/day of ARA for 4 weeks [6], and by 4.3% in adult males given 838 mg/day of ARA for 4 weeks [23]. In the present study, supplementation with 240 and 720 mg/day of ARA increased ARA content by 2.54% and 5.56%, respectively (Figure 2), which was consistent with these findings. These results suggest that age or gender of participants has little effect of ARA supplementation on the increase in plasma ARA content. The results also suggest that the plasma ARA content increases dose-dependently with ARA up to at least around 800 mg/day. The ARA content in plasma phospholipids increased at 2 weeks and was almost the same between at 2 weeks and 4 weeks. The elevated ARA content decreased to almost the initial level during the 4-week washout period regardless of an intake of 240 mg/day or 720 mg/day of ARA. These results were similar to findings seen during intake of 838 mg/day of ARA [23]. The intake of ARA caused a rapid increase in plasma ARA levels but more than 2 weeks of supplementation did not result in any further increases. This is different from DHA, because the velocity of both increases and decreases in plasma DHA content seems slower when fish oil is administered and discontinued during a washout period [32]. The changes in the other fatty acids were also characteristic since plasma DHA and EPA contents remained unchanged throughout the period. The relatively high intake of DHA+EPA (approximately 1 g/day) in the present study (Table 3) might also have contributed to maintaining plasma DHA and EPA levels. The plasma LA content changed in parallel with the plasma ARA content (Figure 2). A previous study also noted this phenomenon [23]. Although ARA and DHA are considered to compete against each other, ARA intake does not reduce plasma DHA content, whereas intake of DHA and EPA reduces both plasma ARA and LA contents [32,33]. The specificity of incorporation into plasma phospholipids from dietary fatty acids seems to be in the order of DHA, EPA > ARA > LA. The reason for the difference between ARA and DHA incorporation is unclear, but the specificity of some enzymes associated with acylation and/or deacylation of each fatty acid might be involved.

Platelet aggregation remains unaffected by an ARA intake of 1.5 g/day or 838 mg/day in randomized controlled studies [21,23]. The present study found that parameters of the coagulation system (PT, APTT and ATIII) remained unaltered and within the normal range. Parameters of chronic inflammation such as plasma hs-CRP, TNFα and IL-6, which are risk factors for cardiovascular disease, remained unchanged, as did plasma adiponectin, which is thought to reduce the risk for cardiovascular diseases. These results suggest that ARA intake does not affect the risk for cardiovascular disease. The parameters for inflammatory diseases (CRP, TNFα and IL-6) and allergic diseases (IgE and eosinophil) were similarly unchanged, suggesting that ARA intake does not evoke inflammatory or allergic diseases. Furthermore, general blood biochemical and hematological parameters remained within normal ranges (Additional file 1, Table S1). Thus, ARA intake appears to be safe under the conditions described here.

In this study, we measured TXA_2_, PGI_2_, PGE_2 _and LXA_4_. TXA_2 _causes platelet aggregation and vasoconstriction, which are considered to lead to cardiovascular disease, PGI_2 _competes against TXA_2 _and suppresses cardiovascular disease and PGE_2 _has various physiological roles, one of which is an inflammatory trigger in addition to possible involvement in cancer growth. LXA_4 _has effects opposite to PGE_2_, and reduces inflammation and cancer growth [34]. Considering their association with diseases, we estimated TXA_2 _and PGI_2 _production as urinary 11-dehydro TXB_2 _and 2,3-dinor-6-keto PGF_1α_, respectively. The reported urinary concentration of 11-dehydro TXB_2 _is 1.489 ng/mg Cre in patients with heart failure, 0.632 ng/mg Cre in those with ischemic heart disease, 0.44 ng/mg Cre in healthy controls [15] and around 0.6 ng/mg Cre in patients with essential hypertension and retinopathy [16]. The mean concentration of 11-dehydro TXB_2 _throughout the present study was < 0.4 ng/mg Cre, which was lower than the levels in these patients and did not significantly differ among the groups. Urinary 2,3-dinor-6-keto PGF_1α _was similarly unchanged and did not differ significantly among the groups. These results show that an increase in the ARA content of plasma phospholipids from 8% to 14% did not affect TXA_2 _and PGI_2 _contents. This is consistent with the finding that parameters for cardiovascular disease did not change. However, these findings seem to differ from those of a previous study in which both urinary 11-dehydro TXB_2 _and 2,3-dinor-6-keto PGF_1α _were slightly increased by intake of 1.5 g ARA/day for 50 days [22]. The larger dose and longer study duration might explain the discrepancies between that study and ours, but the actual reason for the difference remains unclear.

Several reports have described that plasma PGE_2 _increases in inflammatory diseases. For instance, the plasma PGE_2 _concentration increases to > 40 pg/mL in patients with ulcerative colitis [17], and to 54.5 pg/mL in patients with advanced periodontitis [18] compared with about 10 pg/mL in controls. The plasma PGE_2 _concentration in the present study was about 10 pg/mL, which was below the values associated with inflammatory diseases. This value did not change or significantly differ throughout the study. Urinary concentrations of tetranor-PGEM, another marker of PGE_2 _production, are higher in patients with cancer. The reported level is 11.6 ng/mg Cre in patients with colorectal cancer and 7.0 ng/mg Cre in matched controls [19]. Another study indicated a urinary tetranor-PGEM concentration of 15.0 ng/mg Cre in patients with colorectal cancer and 7.17 ng/mg Cre in polyp-free controls [35]. The mean concentration of tetranor-PGEM in the present study was 8 ng/mg Cre in the placebo and the low-ARA group, and 14 ng/mg Cre in the high-ARA group which seems relatively higher compared with normal levels reported previously. The high level of tetranor-PGEM in the high-ARA group was due to the three participants with levels > 30 ng/mg Cre. The higher levels in these participants were not changed by ARA supplementation or during the washout period. Thus, tetranor-PGEM concentration was not significantly changed by ARA supplementation, indicating that ARA intake does not affect a candidate marker of colorectal cancer. Plasma LXA_4 _in the high-ARA groups was significantly increased after the washout period (Figure 4E). It was considered to be unrelated to ARA supplementation because the increase was also observed in the placebo group. Plasma LXA_4 _level may be more variable compared to the other metabolites, but the details are unclear. The increase was slight and considered not to affect the state of the participants.

Next, we analyzed correlations between plasma ARA content and ARA metabolites concentrations after 4 weeks of ARA administration (Figure 5). Concentrations of each ARA metabolite were distributed across a wide range, although all participants were healthy volunteers. None of the values correlated with plasma ARA content or ARA dose. Several individual values were high, but these values seemed normal for these patients, because the high levels in those participants were not changed at baseline and at 2 and 8 weeks. These results indicate that the increase in plasma ARA content from 8% to 14% does not increase the production of TXA_2_, PGI_2_, PGE_2 _and LXA_4_. The production of lipid mediators is not determined primarily by ARA content and seems to be controlled by other factors.

Diet was assessed to ensure that dietary intake of ARA and related fatty acids did not differ among the groups or as a result of the intervention. Participants consumed 170-200 mg/day of ARA from daily meals (Table 3), which is within the normal reported range [1-3] and values did not differ among the groups or with time. The intakes of DHA and EPA were 300-500 mg/day and 500-800 mg/day, respectively, and also did not differ among the groups. Although these values are within the common range in Japan, they are much more than those in Western countries ([2], [36]). Energy and macronutrient intake did not differ among the groups (Table 3), and did not seem to affect the present data.

The effects of ARA supplementation on healthy elderly were clarified here, but studies of patients with specific diseases are needed. In summary, blood parameters of cardiovascular, inflammatory and allergic diseases, as well as urinary and plasma ARA metabolites did not change in Japanese healthy elderly participants who consumed ARA-enriched oil (240 or 720 mg/day of ARA) for 4 weeks although plasma ARA levels significantly increased.

## Competing interests

SK, YI, NT, CH, HT, MK, HK, YS, YK and HS are employees of Suntory Wellness Ltd. or Suntory Business Expert Ltd., which is a manufacturer of foods including ARA-enriched edible oil. IM has consultancy relationships with Suntory Wellness Ltd.

## Authors' contributions

SK participated in the study design, measured lipid mediators and drafted the manuscript. YI and NT participated in the study design and acquired data. CH carried out the nutrition survey. HT and MK measured blood fatty acids. HK participated in the study design and drafted the manuscript. YS, YK and HS participated in the study design and helped to interpret the findings. IM participated in the study design and reviewed the manuscript. All authors read and approved the final manuscript.

## Supplementary Material

Additional file 1 **Supplemental Table S1**. Physiological parameters and blood biochemical and hematological parameters Click here for file
