# Supplementary material for: Supplementation of arachidonic acid-enriched oil increases arachidonic acid contents in plasma phospholipids, but does not increase their metabolites and clinical parameters in Japanese healthy elderly individuals: a randomized controlled study
Source: Lipids Health Dis. 2011 Dec 22;10:241. doi: 10.1186/1476-511X-10-241 (PMC3314585; doi:10.1186/1476-511X-10-241)
Supplement: Additional file 1 — Supplemental Table S1. Physiological parameters and blood biochemical and hematological parameters [file 1476-511X-10-241-S1.DOC]

**Table S1 Physiological parameters and blood biochemical and hematological parameters^1^**

| Parameters^2^ |  | Group | Supplementation | | | Washout |
| --- | --- | --- | --- | --- | --- | --- |
|  |  |  | 0w | 2w | 4w | 8w |
| *Physiological parameters* | | | | | | |
| Body weight | kg | Placebo | 55.7±9.7 | 55.7±9.7 | 55.7±9.6 | 56.0±9.5 |
|  |  | Low-ARA | 56.7±8.4 | 56.9±8.5 | 56.9±8.7 | 57.2±8.6* |
|  |  | High-ARA | 58.8±11.6 | 59.0±11.4 | 58.9±11.4 | 59.0±11.4 |
| BMI | kg/m^2^ | Placebo | 21.6±2.5 | 21.6±2.5 | 21.6±2.4 | 21.7±2.3 |
|  |  | Low-ARA | 22.5±2.0 | 22.6±2.1 | 22.6±2.1 | 22.7±2.1 |
|  |  | High-ARA | 22.5±3.2 | 22.6±3.2 | 22.6±3.1 | 22.6±3.2 |
| Pulse rate | bpm | Placebo | 70±12 | 73±10 | 72±10 | 72± 9 |
|  |  | Low-ARA | 71±11 | 73±13 | 73±11 | 71±10 |
|  |  | High-ARA | 72±9 | 76±10 | 72±10 | 73±10 |
| *Blood biochemical parameters* | | | | | | |
| TP | g/dL | Placebo | 7.1±0.4 | 7.1±0.3 | 7.1±0.4 | 7.1±0.4 |
|  |  | Low-ARA | 7.2±0.3 | 7.1±0.2 | 7.1±0.3 | 7.1±0.3 |
|  |  | High-ARA | 7.2±0.3 | 7.3±0.3 | 7.1±0.3* | 7.2±0.4 |
| ALB | g/dL | Placebo | 4.5±0.2 | 4.4±0.2 | 4.3±0.3** | 4.4±0.3 |
|  |  | Low-ARA | 4.5±0.2 | 4.4±0.2 | 4.3±0.2** | 4.4±0.2* |
|  |  | High-ARA | 4.5±0.1 | 4.4±0.1** | 4.3±0.2** | 4.4±0.2** |
| T-BIL | mg/dL | Placebo | 0.79±0.29 | 0.78±0.29 | 0.79±0.30 | 0.73±0.22 |
|  |  | Low-ARA | 0.87±0.36 | 0.88±0.32 | 0.85±0.35 | 0.83±0.26 |
|  |  | High-ARA | 0.82±0.29 | 0.81±0.28 | 0.90±0.29 | 0.79±0.24 |
| ALP | IU/L | Placebo | 227±82 | 213±68** | 218±70* | 227±70 |
|  |  | Low-ARA | 226±54 | 215±56 | 224±56 | 238±51 |
|  |  | High-ARA | 212±61 | 204±55 | 208±60 | 215±57 |
| AST | IU/L | Placebo | 21±4 | 20±4 | 20±3 | 21±4 |
|  |  | Low-ARA | 21±4 | 21±4 | 21±3 | 23±8 |
|  |  | High-ARA | 22±3 | 20±3 | 22±4 | 22±3 |
| ALT | IU/L | Placebo | 18±7 | 17±6 | 16±4* | 17±6 |
|  |  | Low-ARA | 19±10 | 20±11 | 18±10 | 22±13 |
|  |  | High-ARA | 20±6 | 20±5 | 21±7 | 19±5 |
| LDH | IU/L | Placebo | 186±32 | 190±33 | 186±30 | 184±24 |
|  |  | Low-ARA | 196±24 | 193±26 | 193±25 | 190±25 |
|  |  | High-ARA | 203±26 | 197±25 | 198±20 | 192±21* |
| γ-GTP | IU/L | Placebo | 28±26 | 27±26 | 26±21* | 26±18* |
|  |  | Low-ARA | 29±25 | 30±27 | 28±24 | 32±25 |
|  |  | High-ARA | 25±12 | 23±10 | 29±21 | 24±14 |
| CPK | IU/L | Placebo | 111±39 | 112±40 | 103±33 | 109±35 |
|  |  | Low-ARA | 118±55 | 123±54 | 118±50 | 131±70 |
|  |  | High-ARA | 131±64 | 116±40 | 130±61 | 118±53 |
| T-CHO | mg/dL | Placebo | 223±28 | 227±30 | 218±29 | 220±25 |
|  |  | Low-ARA | 218±34 | 220±31 | 214±34 | 216±33 |
|  |  | High-ARA | 218±29 | 219±26 | 215±30 | 216±33 |
| TG | mg/dL | Placebo | 120±41 | 112±45 | 97±36* | 87±29** |
|  |  | Low-ARA | 111±58 | 96±44 | 99±50 | 96±57 |
|  |  | High-ARA | 85±35 | 91±70 | 74±34 | 86±52 |
| HDL-CHO | mg/dL | Placebo | 59±14 | 62±18* | 62±16* | 64±19** |
|  |  | Low-ARA | 62±15 | 64±16* | 64±17* | 66±16** |
|  |  | High-ARA | 65±15 | 67±16 | 67±15 | 67±14 |
| LDL-CHO | mg/dL | Placebo | 134±24 | 137±22 | 133±24 | 128±23 |
|  |  | Low-ARA | 128±27 | 129±25 | 123±24 | 122±26 |
|  |  | High-ARA | 127±29 | 121±27* | 121±28* | 120±30* |
| GLU | mg/dL | Placebo | 93±9 | 93±9 | 93±10 | 90±9** |
|  |  | Low-ARA | 94±6 | 94±7 | 94±6 | 93±9 |
|  |  | High-ARA | 95±10 | 95±11 | 96±12 | 94±13 |
| PL | mg/dL | Placebo | 235±28 | 239±29 | 228±29 | 231±26 |
|  |  | Low-ARA | 236±31 | 230±30 | 228±31 | 232±33 |
|  |  | High-ARA | 229±25 | 233±23 | 230±30 | 229±28 |
| BUN | mg/dL | Placebo | 13.7±3.5 | 13.8±3.3 | 13.8±3.3 | 13.9±3.0 |
|  |  | Low-ARA | 15.0±3.5 | 14.6±3.6 | 13.3±3.1**^,#^ | 13.9±3.0* |
|  |  | High-ARA | 14.4±2.5 | 14.6±3.8 | 13.1±2.9* | 14.1±2.5 |
| CRE | mg/dL | Placebo | 0.72±0.17 | 0.71±0.17 | 0.68±0.16* | 0.69±0.17 |
|  |  | Low-ARA | 0.68±0.14 | 0.67±0.18 | 0.65±0.15* | 0.67±0.15 |
|  |  | High-ARA | 0.69±0.15 | 0.69±0.16 | 0.66±0.16* | 0.70±0.17 |
| UA | mg/dL | Placebo | 4.9±1.4 | 5.1±1.5 | 5.0±1.3 | 4.7±1.4 |
|  |  | Low-ARA | 4.9±1.3 | 5.0±1.6 | 4.8±1.2 | 4.7±1.3 |
|  |  | High-ARA | 4.8±0.9^#^ | 4.6±0.9 | 4.6±0.9 | 4.7±0.9 |
| Na | mEq/L | Placebo | 140±2 | 141±1** | 141±1** | 141±1** |
|  |  | Low-ARA | 139±3 | 141±1** | 141±2** | 141±1** |
|  |  | High-ARA | 139±3 | 141±1** | 141±1** | 141±1** |
| K | mEq/L | Placebo | 4.44±0.50 | 4.38±0.33 | 4.55±0.43 | 4.51±0.37 |
|  |  | Low-ARA | 4.41±0.35 | 4.50±0.37 | 4.56±0.44* | 4.63±0.48** |
|  |  | High-ARA | 4.37±0.32 | 4.40±0.30 | 4.49±0.40 | 4.70±0.56** |
| Cl | mEq/L | Placebo | 102±1 | 102±1* | 103±1** | 103±1** |
|  |  | Low-ARA | 102±2 | 103±1 | 103±2* | 103±1* |
|  |  | High-ARA | 101±2 | 102±1* | 103±1** | 103±1** |
| Ca | mg/dL | Placebo | 9.62±0.34 | 9.36±0.45** | 9.53±0.36 | 9.45±0.30* |
|  |  | Low-ARA | 9.50±0.27 | 9.39±0.23 | 9.45±0.25 | 9.45±0.28 |
|  |  | High-ARA | 9.59±0.23 | 9.39±0.29** | 9.49±0.25 | 9.51±0.29 |
| *Hematological parameters* | | | | | | |
| WBC | 10^3^/μL | Placebo | 4.92±1.49 | 4.81±1.13 | 5.22±1.74 | 5.19±1.34 |
|  |  | Low-ARA | 4.80±1.13 | 4.94±1.57 | 5.17±1.44 | 5.14±1.27 |
|  |  | High-ARA | 4.73±1.18 | 5.01±1.42 | 5.75±1.90** | 5.26±1.36 |
| RBC | 10^4^/μL | Placebo | 437±39 | 433±35 | 433±39 | 442±45 |
|  |  | Low-ARA | 434±29 | 420±41** | 426±32 | 436±37 |
|  |  | High-ARA | 445±45 | 433±49** | 434±45** | 444±47 |
| HGB | g/dL | Placebo | 13.1±1.4 | 13.1±1.3 | 13.1±1.5 | 13.4±1.6** |
|  |  | Low-ARA | 13.1±0.8 | 13.0±1.1 | 13.1±0.9 | 13.4±0.9* |
|  |  | High-ARA | 13.4±1.3 | 13.3±1.4 | 13.3±1.3 | 13.6±1.3 |
| HCT | % | Placebo | 40.5±4.2 | 40.5±3.4 | 40.2±4.0 | 41.1±4.2 |
|  |  | Low-ARA | 41.1±2.6 | 40.0±3.7* | 40.4±2.7 | 41.5±2.9 |
|  |  | High-ARA | 41.5±3.7 | 40.9±4.1 | 40.9±4.2 | 41.7±4.0 |
| MCV | fL | Placebo | 92.9±6.6 | 93.7±6.7* | 93.3±7.3 | 93.4±7.2 |
|  |  | Low-ARA | 94.9±4.7 | 95.3±4.4 | 95.1±4.1 | 95.6±4.1* |
|  |  | High-ARA | 93.4±3.9 | 94.8±4.0**^,$^ | 94.4±4.1** | 94.3±3.8** |
| MCH | pg | Placebo | 30.1±2.5 | 30.4±2.5* | 30.3±2.7* | 30.5±2.6** |
|  |  | Low-ARA | 30.3±1.4 | 31.1±1.6**^,#^ | 30.8±1.4** | 30.8±1.4** |
|  |  | High-ARA | 30.2±1.6 | 30.7±1.5** | 30.6±1.6** | 30.6±1.5** |
| MCHC | % | Placebo | 32.4±0.9 | 32.4±0.8 | 32.5±0.7 | 32.6±0.8 |
|  |  | Low-ARA | 32.0±0.6 | 32.7±0.5**^,#^ | 32.4±0.5** | 32.3±0.6** |
|  |  | High-ARA | 32.3±0.8 | 32.4±0.7^$^ | 32.5±0.7 | 32.5±0.8 |
| PLT | 10^4^/μL | Placebo | 22.5±4.2 | 23.4±4.3 | 23.4±4.6 | 24.7±5.3** |
|  |  | Low-ARA | 22.5±4.1 | 22.8±4.3 | 22.8±4.8 | 24.5±5.4** |
|  |  | High-ARA | 21.8±3.7 | 22.2±4.0 | 22.2±4.3 | 23.6±3.9** |
| NEUT | % | Placebo | 61±8 | 64±6 | 63±8 | 62±10 |
|  |  | Low-ARA | 60±10 | 62±8 | 62±7 | 61±9 |
|  |  | High-ARA | 57±9 | 61±9 | 60±9 | 59±9 |
| BASO | % | Placebo | 0.3±0.6 | 0.4±0.6 | 0.4±0.8 | 0.3±0.5 |
|  |  | Low-ARA | 0.5±0.7 | 0.6±0.9 | 0.3±0.6 | 0.3±0.6 |
|  |  | High-ARA | 0.4±0.6 | 0.4±0.7 | 0.1±0.3 | 0.3±0.6 |
| MONO | % | Placebo | 4.8±1.2 | 4.9±1.6 | 4.3±1.2 | 5.3±1.4 |
|  |  | Low-ARA | 4.8±1.3 | 4.9±1.2 | 4.7±1.0 | 5.0±1.4 |
|  |  | High-ARA | 5.2±1.5 | 5.4±1.4 | 5.5±1.0 | 5.1±1.5 |
| LYMPH | % | Placebo | 31±8 | 29±6 | 30±7 | 31±9 |
|  |  | Low-ARA | 31±9 | 29±8 | 29±6 | 30±7 |
|  |  | High-ARA | 35±9 | 31±9 | 31±8 | 32±8 |

^1^Values are means ± SD (n = 20, placebo group; n = 22, low-ARA; n = 22, high-ARA). * *p*< 0.05, ***p* < 0.01 versus baseline in the group (ANOVA and Dunnett’s test). ^#^*p* < 0.05 versus placebo group and ^$^*p*<0.05 versus low-ARA group in the case that the amount of change from baseline is significantly different in each time point (ANOVA and Tukey-Kramer test).

^2^TP, total protein; ALB, albumin; T-Bil, total bilirubin; ALP, alkaline phosphatase; AST, aspartate aminotransferase; ALT, alanine aminotransferase; LDH, lactate dehydrogenase; γ-GTP, γ-glutamyl transpeptidase; CPK, creatine phosphokinase; T-CHO, total cholesterol; TG, triglycerides; LDL-CHO, low-density lipoprotein cholesterol; GLU, glucose; PL, phospholipids; BUN, blood urea nitrogen; CRE, creatinine; UA, uric acid; WBC, white blood cells; RBC, red blood cells; HGB, hemoglobin; HCT, hematocrit. MCV, mean cell volume; MCH, mean cell hemoglobin; MCHC, mean cell hemoglobin concentration; PLT, platelets; NEUT, neutrophils; BASO, basophils; MONO, monocytes; LYMPH, lymphocytes.
